# ^18^F-FDG PET CT in cardiac device infections - A Case series

**DOI:** 10.22038/AOJNMB.2024.77504.1547

**Published:** 2024

**Authors:** Awiral Saxena, Manjit Sarma, Bhagirath Bhad, Ramkesh Ratheesan, Padma Subramanyam, P. Shanmuga Sundaram

**Affiliations:** Department of Nuclear Medicine & Molecular Imaging, Amrita Institute of Medical Science, Kochi, Kerala, India

**Keywords:** ^18^F-FDG PET CT, Coronary Stent infection, Prosthetic valve infection, CIED infection, Infection imaging  A B S T R A C T

## Abstract

With the increasing number of interventional cardiology procedures, the number of cardiac device infections (including pacemakers, prosthetic valves, coronary and aortic stents) have also increased. These infections can cause significant morbidity and can even lead to mortality if not managed promptly. If suspected clinically the first-line imaging modality is Trans-Thoracic Echocardiography, while Transesophageal Echocardiography is also used in selected cases. The confirmation of a cardiac device infection is mostly done with the help of blood or pus culture. Even though Echocardiography is a very efficient technique for the evaluation of the heart, it cannot differentiate infection from thrombus or fibrosis. With the increasing availability of Positron Emission Tomography CT (PET CT) machines worldwide, the use of ^18^F-FDG PET CT for infection imaging has gained traction, especially for cardiac device infection. Most of the recent studies show a good diagnostic accuracy of ^18^F-FDG PET CT with many of the recent diagnostic and management guidelines now acknowledging its role, especially in equivocal cases. We present six such cases where ^18^F-FDG PET CT provided valuable information either for diagnosis, confirming the presence of infection, delineating extent, therapy response or sometimes even helping appropriate treatment decision making in patients with suspected cardiac device infection.

## Introduction

 More than 1 million cardiac pacemakers are implanted every year worldwide (1), with more than 20,000 pacemakers being implanted each year in India alone (2). Similarly, other cardiac interventions have also gained pace and are growing at rapid rates. In India, more than four hundred thousand percutaneous coronary intervention (PCI) procedures are performed annually with more than half a million stents being placed (3). With the increasing number of interventions, the cases of infections are also increasing. Cardiac stent and implant infections are morbid, costly, and difficult to manage (4-7).

 Diagnosis of cardiac device infection is a major challenge, as many patients exhibit only vague symptoms (8, 9). There is no international consensus on the diagnosis and management of cardiac device infections but guidelines such as Expert Consensus Statement on Lead Extractions (2009), (10) an American Heart Association scientific statement (2010) (11) and a British guideline paper (2015) (12) are useful for guiding decisions. More recently in 2017, Heart Rhythm Society released an expert consensus statement on cardiovascular implantable electronic device lead management and extraction (13). The first-line imaging tests for diagnosing probable endocarditis are transthoracic (TTE) and transesophageal (TEE) echocardiograms. Echocardiography, however, presents greater difficulties in patients who have implanted cardiac devices. When evaluating peri-implant soft tissue, such as abscesses, fistulas, pseudoaneurysms etc. which are crucial for surgical planning and linked to unfavourable surgical outcomes, cardiac computed tomography (CT) or cardiac CT angiography (CTA) adds value to transverse endovascular ultrasound (TEE) or transthoracic echocardiography (TEE) (14). However, neither are specific for the presence of active infection and are also affected by artifacts caused by implanted device.

^18^F-fluoro-2-deoxyglucose (FDG) targets the inflammatory cells (macrophages, neutrophils, and lymphocytes) at the site of infection/ inflammation and imaging the ^18^F-FDG signal with positron emission tomography (PET) is not affected by device artefacts, when non attenuation-corrected images are included in the assessment. FDG PET imaging offers the extra benefit of detecting inflammation early in the infection phase (15) and is also useful in response assessment. We present six such cases of cardiac device infections which were evaluated with FDG PET CT in our institution.

## Cases:

***Case 1***

 A 54-year-old man had presented with fever and chills in June 2023 with a history of prosthetic mitral valve placement 6 months ago. 

**Figure 1:**
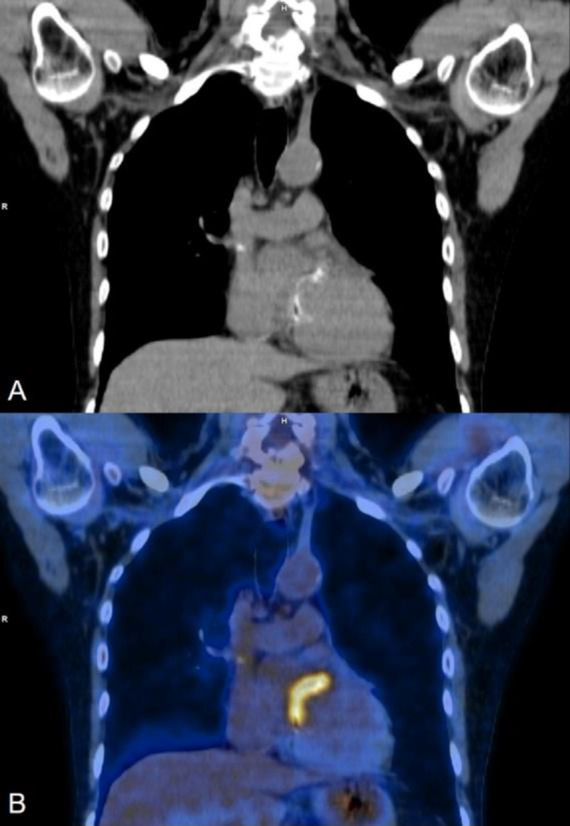
Coronal section (**A**) CT and (**B**) fused FDG PET CT images showing intense FDG uptake (SUV_Max_: 6.1) along the prosthetic mitral valve

 He was on empirical antibiotics elsewhere prescribed from another hospital, but the fever had persisted. His C-reactive protein (CRP) was 50 mg/L (Normal range 0-1 mg/L). Trans-esophageal Echocardiography showed no vegetation. He was started on Tab Fluconazole as his blood culture & sensitivity results showed the presence of Candida parapsilosis which was sensitive to fluconazole and his inflammatory markers showed a downtrend with CRP 28 mg/L. Due to persistent fever, he underwent a whole-body FDG PET CT scan, which revealed increased FDG uptake in the prosthetic mitral valve (SUV_Max_: 6.1), suggesting an active infection (Figure 1). SUV or Standardized Uptake Value is an important semi-quantitative parameter of PET imaging. It is the ratio of the activity in tissue per milliliter of the injected activity divided by the patient’s body weight in kilograms and measured as grams/milliliter. He was then started on treatment of prosthetic valve endocarditis, suspecting fungal endocarditis, with Inj. Micafungin and Teicoplanin for four weeks, which was then further continued for four more weeks. After the completion of treatment, he underwent another FDG PET CT scan at a different institute (images unavailable) which showed a complete resolution of the metabolic activity along with normalization of the inflammatory markers. 

 FDG PET CT in this case was helpful in localizing the infection focus and assessing the treatment's efficacy.

***Case 2***

**Figure 2:**
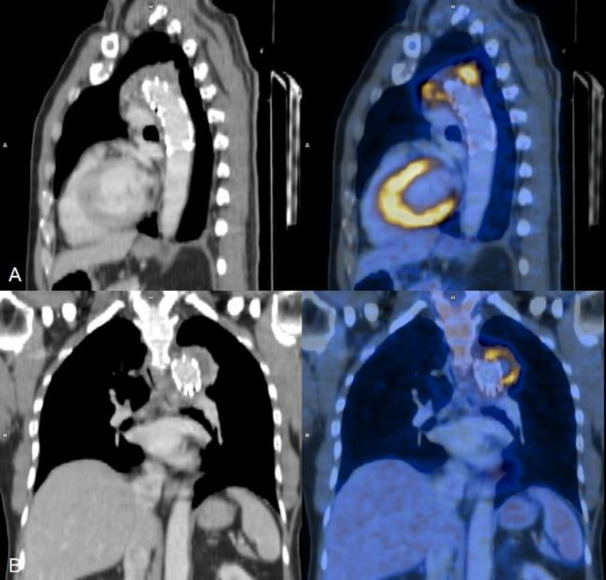
(**A**) Saggital and (**B**) Coronal section CT and fused FDG PET CT (left to right) images showing abnormal increased FDG uptake (SUV_Max_: 10.1) in aortic wall aneurysm in relation to the EVAR stent

 A 71-year-old man had presented with a contained rupture of post subclavian aortic aneurysm (diagnosed on CT chest) along with recurrent fever since 9 months with left sided chest pain. In view of his evening rise of fever and with past evaluation not ruling out TB while all other culture and serological tests were negative, he was initiated on empirical Anti Tubercular Treatment (ATT), resulting in symptomatic improvement. Due to an imminent rupture, he underwent an Endovascular Aortic Aneurysm Repair (EVAR) procedure in December 2015, which involved the placement of a thoracic stent graft system. He was started on intravenous ceftriaxone and continued with anti-tubercular therapy. He experienced a recurrence of fever, prompting him to get a whole-body FDG PET CT scan. The scan revealed significantly increased FDG uptake (SUV_Max_: 10.1) at the location of the damaged aortic wall, in connection with the endovascular stent (Figure 2). Suspecting persistence of active infection, he was subsequently switched to intravenous meropenem administration. It was decided to continue the extended spectrum of antibiotics and the plan was to proceed with aortic repair and abscess evacuation if there was no satisfactory response. However, the patient was lost to further follow-up. FDG PET CT helped in confirming that the infection was indeed localized to the aneurysmal site and even though a pre-treatment baseline FDG PET CT was not done, the high SUV_Max_ confirmed the persistence of active infection which was likely unresponsive to the empirical therapy.

***Case 3***

 A 42-year-old woman with Triple Vessel Disease (TVD), underwent a PTCA (Percutaneous Transluminal Coronary Angioplasty) to proximal Right Coronary Artery (RCA) and Plain Old Balloon Angioplasty (POBA) to obtuse marginal branch of left circumflex artery (LCx) in August 2023. A month later she had an acute STEMI, following which she underwent another PTCA with placement of a cover stent in view of an aneurysm at the proximal part of previous RCA stent. She developed fever with cough four days later. 

**Figure 3:**
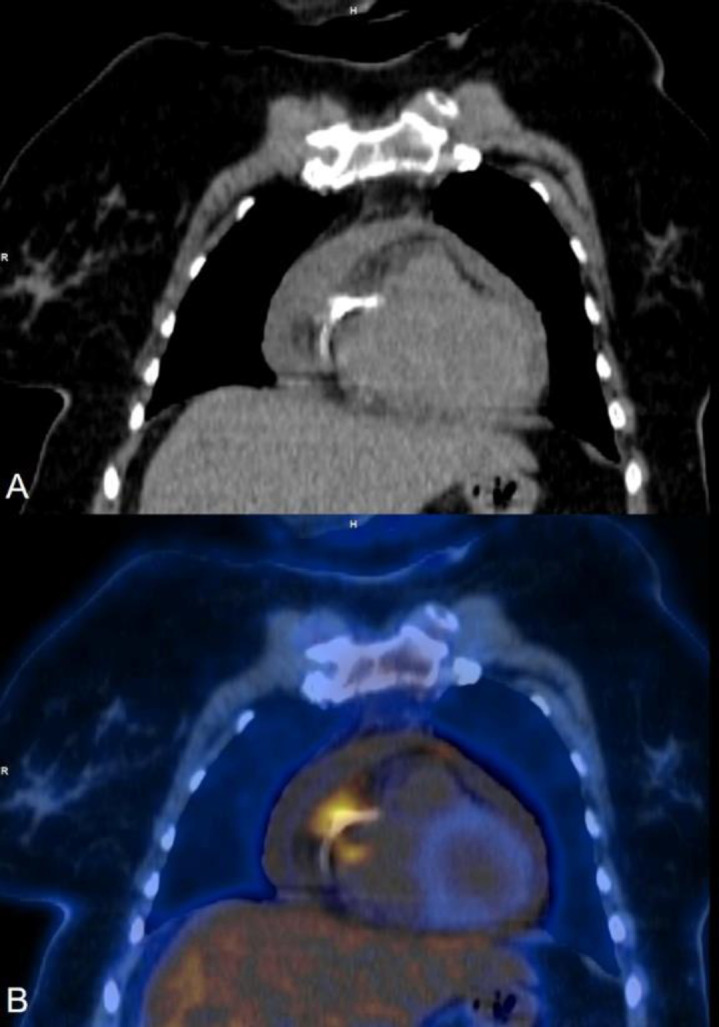
Coronal section (**A**) CT and (**B**) fused FDG PET CT images showing focal abnormal increased FDG uptake (SUV_Max_: 4.5) along the RCA stent

 Given the presence of aneurysm at proximal part of RCA she was advised a regional FDG PET CT scan. It showed focal FDG uptake along the RCA stent (SUV_Max_: 4.5) (Figure 3) with diffuse minimal FDG uptake (SUV_Max_: 2.6) in moderate pericardial effusion. She was started on Intravenous Vancomycin and Amikacin and improved symptomatically along with reduction in inflammatory markers (CRP 85 mg/L from 110 mg/L). She wished to continue treatment at her local hospital and has not come for follow-up since. FDG PET CT was useful for confirming the clinically suspected focus of infection in this case and streamlining management.

***Case 4***

 A 73-year-old man underwent PTCA to the left anterior descending artery (LAD) at another hospital in March 2023 and presented to our institution after 10 days with chest pain and fever. Blood and urine cultures were negative with raised CRP (193 mg/L) and Total Leucocyte counts (21400/uL). The patient had bilateral mild pleural effusion on CT chest and mild pericardial effusion, which was increasing on serial transthoracic echocardiography (TTE). Pleural fluid cultures were also negative. 

**Figure 4:**
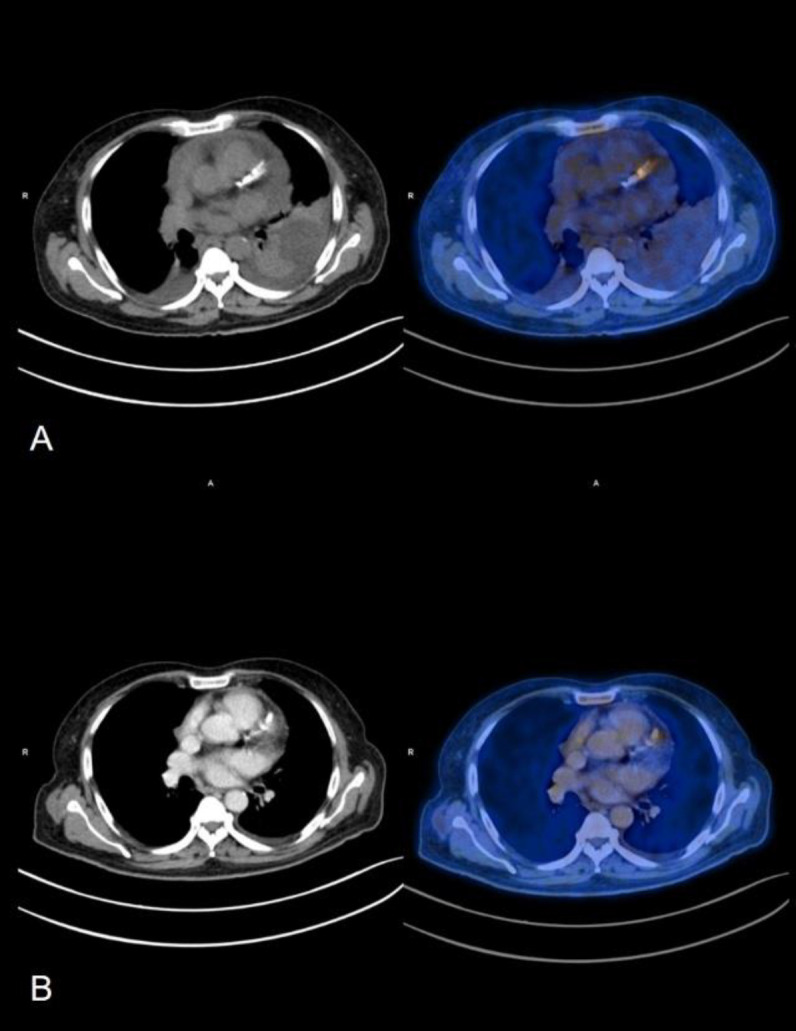
Transaxial CT and Fused FDG PET CT images (left to right); (**A**) showing abnormal increased FDG uptake (SUV_Max_: 3.5) along the LAD stent along with bilateral pleural effusion and mild pericardial effusion. Follow-up FDG PET CT (**B**) showing significant reduction in intenstiy & extent of FDG uptake along the LAD stent along with near complete resolution of pleural and pericardial effusion

 Regional FDG PET CT (done 15 days after angioplasty) showed linear FDG uptake (SUV_Max_: 3.5) along the coronary stent (Figure 4A) suggesting the possibility of a low-grade infection. He was started empirically on Inj. Meropenem and Teicoplanin, following which he improved clinically, and inflammatory markers also reduced (CRP 6.1 mg/L and TLC 6600/uL). A follow up FDG PET was done after 3 weeks, which showed significant reduction in intensity & extent of FDG uptake (Figure 4B). 

 After the antibiotics were stopped after four more weeks, he did not show any symptoms and has been on follow-up since. In this case, given the timing of the symptoms (post-operative period) and the presence of increasing pericardial effusion, there was a suspicion of coronary stent infection. It highlights the fact that coronary stent infection, albeit a rare post-operative complication, should be considered as a differential in these cases and FDG PET CT can be a valuable tool to evaluate these patients even in immediate post-operative period.

***Case 5***

**Figure 5:**
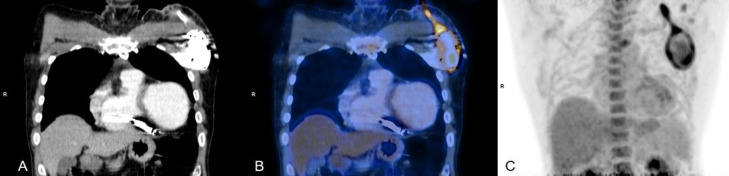
Coronal section (**A**) CT , (**B**) fused FDG PET CT and (**C**) Maximum Intensity Projection (MIP) images showing abnormal increased FDG uptake (SUV_Max_: 6.7) around the pacing box extending to the proximal lead wire

 A 56-year-old man with an implanted Automated Implantable Cardioverter Defibrillator (AICD) (placed in January 2020), developed inflammation along with oozing from at the implantation site in November 2022. He had also developed dyspnea on exertion. A whole-body FDG PET CT was requested to look for the extent of infection at the local site and also to evaluate the rest of the body for any other possible site of infection. The scan showed intense FDG uptake (SUV_Max_: 6.7) at the implantation site in anterior chest wall which was also extending into the proximal part of lead wires (Figure 5). He was treated with IV Vancomycin for 2 weeks and then taken up for explantation. Another AICD was implanted on the opposite side after 1 month and the patient has been doing well since. FDG PET CT helped to assess the extent of infection which can be important in formulating the management plan. Also in this case, a whole body FDG PET excluded the presence of another focus of infection.

***Case 6***

 A 51-year-old woman, a case of dilated cardiomyopathy presented with congestive heart failure and severe LV dysfunction. She is a known diabetic with diabetic nephropathy and hypertensive. She underwent Cardiac Resynchronization Therapy Defibrillator (CRT-D) implantation at our institution. Three months post-surgery she had presented with fever and tiredness for 5 days. 

**Figure 6:**
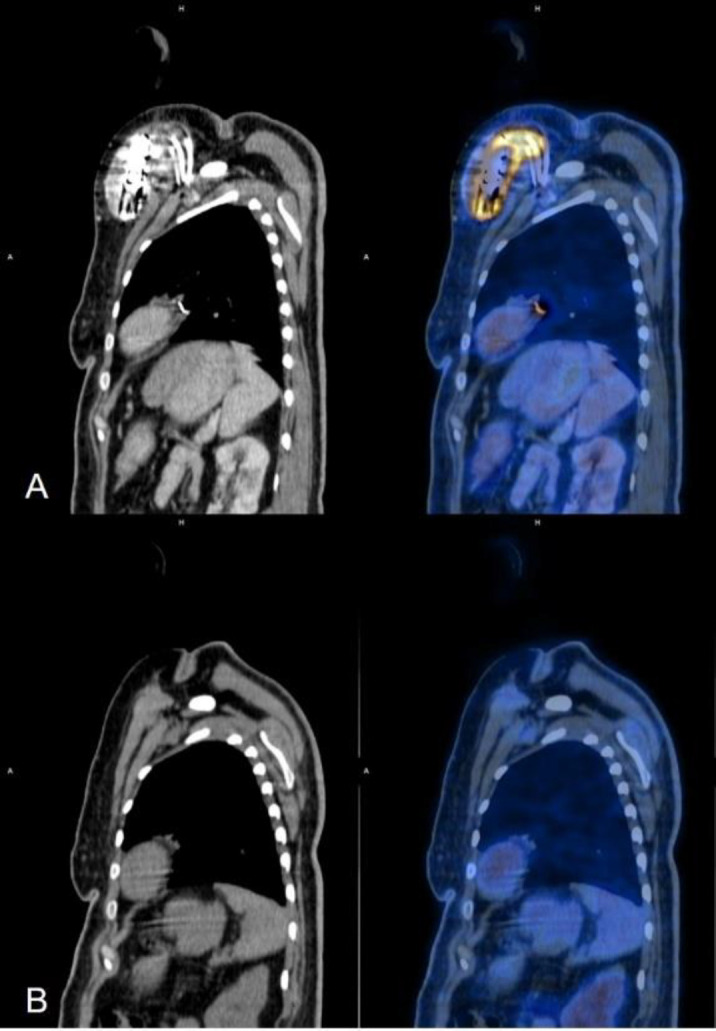
Saggital section CT and fused FDG PET CT images (left to right); (**A**) showing abnormal increased FDG uptake (SUV_Max_: 8.6) in hypodense collection around the pacing box with extension of the FDG uptake along the adjacent leads. (**B**) Follow-up FDG PET CT done 4 months later (post explantation of device) showing no abnormal increased FDG uptake at post-surgical site

 Echocardiography showed dilated left atrium (LA) and left ventricle (LV) with global LV hypokinesia and moderate LV systolic dysfunction. Ultrasonography of the implantation site showed a well-defined anechoic collection surrounding the pacemaker leads in the left axillary region. Suspecting infection, a FDG PET CT was requested which showed intense FDG uptake (SUV_Max_: 8.6) in hypo dense collection around the pacing box with extension of the FDG uptake along the adjacent leads (Figure 6A). The CRT-D was explanted and the pus culture showed the presence of Burkholderia cepacia. He was started on Inj. Meropenem & Tab Levofloxacin. 

 1 month later, Trans-thoracic Echo-cardiography (TTE) showed a 1.1cm filamentous structure with independent mobility in right atrium close to tricuspid valve, diagnosed as tricuspid valve endocarditis and continued on same treatment (as they were sensitive on pus culture) for 4 weeks and stopped. The size of the vegetation decreased minimally in serial echocardiography evaluations and was considered a healed lesion. 

 Three months later a follow up FDG PET CT was done which showed significant reduction in extent & intensity of metabolic activity in left anterior chest wall (Figure 6B). Minimal FDG uptake (SUV_Max_: 2.1) was present in the region, likely due to post-procedural inflammation. 

 Further follow-up TTE did not show presence of any vegetation. Here, FDG PET CT delineated the extent of the infection apart from the suspected site of involvement and given the lead involvement, patient was continued on antibiotics and closely followed up with echocardiography which confirmed the presence of infective endocarditis. FDG PET CT can also be effectively used to see the therapeutic response.

## Discussion

 The incidence of cardiac implantable electronic devices (CIED) infections is rising, retrospective data shows that they range from 0.6% to 6% (4). Because (CIEDs) have both subcutaneous and intravascular components, the term "CIED infection" refers to both subcutaneous and bloodstream infections (4). 

 The European Heart Rhythm Association and the Heart Rhythm Society both have precise definitions for terms like pocket infection and lead-associated endocarditis (13) and have moved away from historically used phrases like device-associated endocarditis. Infections are more common in Implantable Cardioverter Defibrillators (ICD) than pacemakers (16).

 Although coronary stent infection (CSI) is a rare complication of percutaneous coronary intervention (PCI), occurring in less than 0.1% of cases (17), they are associated with a high mortality rate (18). With the relatively high number of PCI being performed in recent years, this has become clinically significant. Drug-eluting stents have a higher risk of infection than bare-metal stents (17).

 The risk of infection increases with implantations in 'higher risk' patients, such as those with diabetes, heart disease, or renal failure (19). Diagnosis is mainly made based on clinical signs, symptoms and echocardiography. 

 Both Trans-Thoracic Echocardiography (TTE) and Trans-Esophageal Echocardiography (TEE) are useful in diagnosis, with TTE providing more information about the ventricular size & function while TEE is more sensitive for detecting aortic and mitral valve endocarditis. 

 Both modalities are not accurate in differentiating thrombus, fibrosis, or infection.

 While previous guidelines (10, 11) had not mentioned FDG PET in evaluation of CIED infection, the 2015 British guideline paper has mentioned FDG PET CT as a modality helpful in diagnosis of CIED but had refrained from recommending it as a routine clinical test. With availability of more evidence, the Heart Rhythm Society 2017 expert consensus statement currently recommends Echocardiography (preferably Trans-esophageal echocardiography) as the 1^st^ line investigation for diagnosing CIED-related endocarditis and/or lead infection, even with cases presenting with only pocket infection, while FDG PET CT is recommended in doubtful cases of lead or pocket infection. While guidance for the diagnosis of CIED infections is provided by different societies, no such guideline is available for CSI. Prosthetic valve endocarditis is a known indication for FDG PET CT and is recommended by the European Society of Cardiology 2015 Guidelines for infective endocarditis for its diagnosis (20).

 For CIED infection evaluation, a meta-analysis showed that FDG-PET/CT had a pooled sensitivity and specificity of 87% and 94%, respectively (21) while sensitivity and specificity for prosthetic valve endocarditis vary between 73 to 96 and 80 to 94 % respectively (22). FDG PET CT has good diagnostic accuracy in coronary stent infections, but due to the lower incidence of stent infections only a few case reports are available and no meta-analysis data is available.

 Cells with elevated glucose transporter expression, such as immune cells in an aseptic postsurgical inflammatory site, can accumulate FDG. It is sometimes difficult to distinguish between generic inflammation and device infection using the FDG uptake pattern and intensity. The European Society of Cardiology guideline (20) states that because nonspecific FDG uptake may be an issue, FDG-PET/CT should not be used for evaluating device infection within three months of surgical implantation. Pizzi et.al, however, has not demonstrated a significant effect of physiological FDG uptake on the precision of device infection diagnosis at various time points following surgery (23), while Sarrazin et.al found only modest FDG activity within two months following surgery and no uptake at two months or later (24). We also had an early post-operative case of stent infection (Case 4) where the focality and pattern of FDG uptake was more useful than the intensity of FDG uptake. The inflammatory FDG uptake can be a problem in cases with low grade infection if there is significant background uptake, but in cases with significant infection FDG PET CT is probably not affected by the time-point of imaging.

 Another factor is physiological myocardial FDG uptake, which acts as a barrier in interpreting these scans and should be suppressed for an optimal scan. Since both glucose and free fatty acids are substrates for myocardial metabolism, the presence of normal physiological uptake (via GLUT-1 and GLUT-4 transporters) due to glucose metabolism is a hindrance and different interventions have been tried to switch the myocardium to near exclusive free fatty acid metabolism. During fasting, the levels of insulin in the bloodstream decrease, and the heart's energy needs are primarily satisfied by fatty acids. The combination of a high-fat low-carbohydrate diet (25) and use of intravascular heparin results in good suppression of myocardial physiological FDG uptake (26). We follow the combination of both protocols along with prolonged fasting (12-18 hours) in our institute for optimal suppression. Visualization of the entire chest with CT and sometimes the entire body with FDG PET/CT gives the added benefit of evaluating infection in the extracardiac components of devices, which could potentially identify the unexpected source of the primary infection and/or lead to discovering embolic consequences of endocarditis in the body, which may have a significant impact on patient management and outcomes (27). In our institution, some of the patients are referred for a whole-body FDG PET CT to look for any other focus of infection, apart from the clinically suspected cardiac device infection.

 Active infection may be difficult to be ruled out, especially in patients on prolonged antibiotic therapy and FDG PET CT can be a useful tool in these cases. Although FDG PET CT is not routinely recommended for treatment response evaluation, it acts as a problem-solving tool in clinically indeterminate or high-risk cases. Large-scale studies are required to confirm the utility and standardize the use of FDG PET CT in response assessment evaluation. 

 FDG PET CT is an effective tool for localizing the site of infection in suspected cardiac device infection and can help in determining the extent and severity of infection. It may also be used for the assessment of treatment response.
